# Alcohol use and its associated factors among Ethiopian military personnel

**DOI:** 10.1136/military-2022-002217

**Published:** 2022-11-25

**Authors:** Alemu Tesfahun Fida, M Kaba, A Worku

**Affiliations:** 1 Public Health, Defense University College of Health Sciences, Addis Ababa, Ethiopia; 2 School of Public Health, Addis Ababa University, Bishoftu 1419, Ethiopia

**Keywords:** audit, epidemiology, substance misuse, public health

## Abstract

**Background:**

Studies indicate that alcohol use is more prevalent in the military and that such use is related to coping mechanisms for stress. Alcohol use could result in health and social problems. However, little is known about alcohol use in Ethiopian military personnel.

**Objective:**

To assess the level of alcohol use and its associated factors in the Ethiopian military.

**Methods:**

A cross-sectional study including participants from the Ground and Air Forces of the Ethiopian National Defence Forces was conducted from February to June 2021. A representative sample of 502 military personnel from the two forces participated in the study. A structured questionnaire was developed to assess the individual level of alcohol use and its associated factors. The Alcohol Use Disorder Identification Test (AUDIT) was used to identify likely problematic alcohol use (AUDIT score ≥8). Hierarchical multivariable logistic regression models were run to identify associated factors with alcohol consumption.

**Results:**

Approximately half of the respondents (49.8%, 95% CI 45.4% to 54.0%) were alcohol drinkers. Of the current alcohol users, 142 (63.1 %) were infrequent users; 60 (26.7 %) were moderate drinkers; and 23 (10.2 %) were heavy drinkers. Based on the AUDIT composite score, 71 (33.0%) of male participants were classified as having a score indicative of hazardous and harmful drinking and possible alcohol dependence behaviours. After adjusting for covariates, alcohol drinking was statistically significantly associated with higher odds of being: male, younger age, part of the Ground Force, smoker and high risk-taker.

**Conclusions:**

This study provides an initial step to addressing patterns of harmful and hazardous alcohol use in the Ethiopian National Defence Forces. Findings indicate the need to integrate alcohol abuse prevention into existing health education and behaviour change efforts of the Ethiopian National Defence Forces.

**WHAT IS ALREADY KNOWN ON THIS TOPIC**

- Alcohol misuse is a common problem in the military.

**WHAT THIS STUDY ADDS**

- Women military personnel were included in this study, which might not have been the case in previous studies in low-income countries.

**HOW THIS STUDY MIGHT AFFECT RESEARCH, PRACTICE OR POLICY**

- The findings highlight the association between smoking, high risk-taking and alcohol may need further attention.

## Introduction

Historically, alcohol use has been used to alleviate stress in the military, which is an unhealthy coping mechanism.^1^ Available evidence suggests that there is an association between combat stress, peer pressure and alcohol misuse among military personnel.^2 3^ Alcohol consumption in the military may also be influenced by societal and cultural factors.^4^ Male-dominated occupations, such as the military, had higher levels of hazardous and harmful alcohol use.^5^ Heavy drinking increases the risk of morbidity and mortality.^6^ In the military, alcohol use was associated with poorer health and social functioning.^7^ The problem might extend to compromising the military’s capacity to carry out its mission and result in reduced readiness, lower force fitness and loss of productivity.^8^

The problem of alcohol use could be reflected by the prevalence of current drinkers. According to the Armed Forces of Sri Lanka study, 71.2% were current drinkers, with 54.8% being infrequent users.^9^ From sub-Saharan African studies, the Armed Forces of Congo found 77.2%^10^; the Angolan military study found close to 60%^11^; and the Nigeria Army study found 53.9% of participants had consumed alcohol.^12^ In the general population of Ethiopia, the prevalence of alcohol drinking for males aged 15–59 was 46.6%, with a 95% CI of 45% to 47%.^13^

The alcohol problems could be assessed using various methods based on the available resources and acceptance. The Alcohol Use Disorder Identification Test (AUDIT) is one of the screening tools for alcohol problems. AUDIT was developed by the WHO in a multinational study of primary healthcare facilities and offers several advantages over alternative assessment tools for our purposes in a military setting, specifically a focus on early identification of hazardous drinking rather than chronic dependence and severe adverse medical consequences.^14^ The AUDIT has been acknowledged as a valid instrument for identifying alcohol misuse or dependence among the Australian military population.^15^

AUDIT scores of more than eight indicate hazardous drinking. The Sri Lanka Armed Forces study had a prevalence of 16.69% hazardous alcohol use.^9^ From sub-Saharan African countries, 24% qualified for the Malawi Defence Force study^16^; 58.9% qualified for the Botswana Defence Force study^17^; nearly 35% qualified for the Angolan soldiers’ study^11^; and 15.8% qualified for the Republic of the Congo Armed Force study^10^ for hazardous alcohol use. The pooled prevalence of hazardous alcohol use from various studies of the Armed Forces was 34%, with a 95% CI of 18% to 52%.^5^ Based on the Global Status Report on Alcohol and Health, the prevalence of alcohol use disorders in the general population of Ethiopia was 2.5% for both sexes.^18^

Most research on alcohol use among military personnel is from high-income countries and may have limited applicability to military personnel of low-income nations due to context differences. The aim of this study was to assess alcohol use and its associated factors among Ethiopian military personnel.

## Methods

### Setting

The study was carried out in the National Defence Forces of Ethiopia.

### Study design and participants

A cross-sectional study was conducted from February to June 2021. The sample size was determined using a single proportion formula, taking the level of significance of 0.05, hazardous drinking prevalence of 15.8%,^10^ marginal error of 5%, design effect of 2 and non-response rate of 5%, which was calculated to be 430. Since this study was a part of a large study, Assessment of the Level of Health Related Behaviours among Ethiopian Military Personnel, the highest sample size (522) was taken. Participants were recruited from two divisions of the Ground Force and two bases of the Air Force, using stratified sampling techniques. Exclusion criteria included being on non-active duty, on annual leave during the study period, being a service academy student and being a health professional.

### Data collection

The questionnaire was administered in person by military personnel with at least a high school graduate. Participants were selected from the units using proportional allocation. Interviews were scheduled at the convenience of the units, close to their duty stations. The interviewers were trained for 2 days on the brief overview and aim of the study, the contents of the questionnaire, data collection techniques and research ethics in brief. Participants were interviewed by data collectors of comparable rank level. In addition, the data collection was supervised by three nurses and a principal investigator.

### Key measures

#### Measurement of alcohol use

Alcohol consumption was assessed using the AUDIT, which identifies hazardous, harmful and dependent alcohol use. People who score 0 are abstainers, while levels of risk are identified by categorical ranges as follows: low risk=a score of 1–7, hazardous drinking=a score of 8–15, harmful drinking=a score of 16–19 and possible alcohol dependence=a score of 20 or greater.^19^

### Drinking-level classifications

The coding for classifications was based on the US 2014 Health Related Behaviours Active Duty Personnel Survey Methodology.^20^ To determine current drinking levels, the number of days the respondent drank was used to calculate the average number of drinks per week, as follows: [(# days per year)(# drinks per year)/365 days]. A ‘current drinker’ was defined as having at least 12 drinks in their lifetime and reporting one or more days of drinking in the past 12 months. Current drinkers were categorised into three levels of drinking intensity as follows. An ‘infrequent/light drinker’ was defined as having less than four drinks per week in the past year. A ‘moderate drinker’ was defined as having 4–14 drinks per week for men and 4–7 drinks per week for women in the past year. A ‘heavy drinker’ was defined as having more than 14 drinks per week for men and more than seven drinks per week for women in the past year.

### Demographic and military characteristics

Demographic variables of interest included age, sex, marital status (single, married, widowed or divorced) and highest education level completed (high school or less, some college and a bachelor’s degree or more). Military characteristics of interest included rank (non-commissioned officer (NCO) and lower ranks and commissioned officer) and force branch (Air Force and Ground Force). Military service year and age at first drink were also considered.

### Cigarette smoking

Cigarette smoking was defined as having smoked at least 100 cigarettes in one’s lifetime and having smoked cigarettes during the past 30 days.^21^

### Depression

The nine-item Patient Health Questionnaire^22^ was used to assess depression. Interpretation of the total score was based on 1–4 as minimal depression, 5–9 as mild depression, 10–14 as moderate depression, 15–19 moderately severe depression and 20–27 as severe depression. Due to small numbers, those with a score of 5 or higher were collapsed into one group.

### Risk-taking propensity

Four survey items were taken for risk-taking, measured on a 5-point scale ranging from ‘not at all’ to ‘a great deal’. To generate a risk-taking propensity scale based on the average of the total responses, a great deal=1, a lot=0.75, somewhat=0.5, a little=0.25, not at all=0. We then dichotomised risk-taking into two composite response categories: those with an average score of 0.75 or greater were taken under ‘high risk-taking’, whereas those with an average score of less than 0.75 were taken under ‘low risk-taking’.^20^

### Data analysis

Frequencies and percentages for categorical variables, mean and SD, median and IQR after checking normality for continuous variables were computed. Bivariate logistic regression models were used to examine associations between demographic and military characteristics with alcohol use. The OR and 95% CIs were calculated using cross-tabulation for the crude OR and hierarchical multivariable logistic regression for the adjusted OR. A p value of <0.25 was used to take variables to multivariable regression. The first regression model contained sociodemographic variables, and the second model added smoking, depression levels and risk-taking propensity. Model adequacy was assessed using the goodness of fit with the Hosmer-Lemeshow test. All data analyses were carried out using SPSS V.24 statistical software. All tests were two-tailed. A p value of <0.05 was considered statistically significant.

## Results

### Sociodemographic characteristics

Table 1 shows detailed sample demographics. The overall response rate was 502 individuals, 96.2% of the eligible sample (522), 89 (17.7%) of whom were female and 413 (82.3%) of whom were male. The median age was 25 years, with an IQR of 22–34 years. About 39.6% had 0–5 years of military experience, and 60.4% had six or more years. The length of military service ranged from 0.5 to 30 years. Four hundred twenty-four (84.5%) of the respondents were Ground Force, and 78 (15.5%) were Air Force.

**Table 1:** Sociodemographic characteristics overall of military personnel

Variable	Category	Respondents, n (n=502)	Per cent
Sex	Female	89	17.7
	Male	413	82.3
Age (years)	18–20	76	15.1
	21–25	175	34.9
	26–35	137	27.3
	36–45	91	18.1
	46+	23	4.6
Religion	Orthodox Christian	300	59.8
	Protestant	128	25.5
	Islam	60	12.0
	Catholic	11	2.2
	Other	3	0.6
Marital status	Single	289	57.6
	Married	209	41.6
	Divorced	4	0.8
Education	High school or less	387	77.1
	Some college	50	10.0
	Bachelor’s degree or more	55	11.0
Military rank	NCO and lower ranks	420	83.7
	Commissioned officer	82	16.3
Force branch	Air Force	78	15.5
	Ground Force	424	84.5

NCO, non-commissioned officer.

### Alcohol use

Half of the 225 respondents (49.8%) with 95% CI (45.4% to 54.0%) were current drinkers. One hundred forty-two (63.1%) were infrequent drinkers; 60 (26.7%) were moderate drinkers; and 23 (10.2%) were heavy drinkers (Table 2).

**Table 2:** Sociodemographic characteristics by drinking level among military personnel

Variable	Category	Light drinker, n (%)	Moderate drinker, n (%)	Heavy drinker, n (%)
Overall (n=225)		142 (63.1)	60 (26.7)	23 (10.2)
Age (years)	18–20	19 (67.9)	4 (14.3)	5 (17.8)
	21–25	48 (61.5)	22 (28.2)	8 (10.3)
	26–34	41 (68.3)	15 (25.0)	4 (6.7)
	35–45	30 (63.8)	12 (25.5)	5 (10.7)
	46 and above	4 (33.3)	7 (58.4)	1 (8.3)
Sex	Female	20 (62.5)	2 (6.2)	10 (31.3)
	Male	122 (63.2)	58 (30.1)	13 (6.7)
Military rank	NCO and lower ranks	126 (64.9)	49 (25.3)	19 (9.8)
	Commissioned officer	16 (51.6)	11 (35.5)	4 (12.9)
Force branch	Air Force	13 (81.3)	1 (6.2)	2 (12.5)
	Ground Force	129 (61.7)	59 (28.2)	21 (10.1)

The mean (SD) age at the first drink was 19.3±4.6 years. The median number of estimated days all respondents drank any type of alcoholic beverage in the past 12 months was 40, with an IQR of 20–96 days.

### Alcohol Use Disorders Identification Test

What stands out in the AUDIT table is the risk levels of drinking across the respondents. Based on their AUDIT composite score, respondents were classified into 167 (66.8%) as low risk (< 8), 69 (27.6%) as hazardous drinking (8-15), 9 (3.6%) as harmful drinking (16-19) and 5 (2.0%) as possible alcohol dependence (≥20) (Table 3).

**Table 3:** Sociodemographic characteristics by AUDIT composite score among military personnel (n=250)

Variable	Category	Low-risk alcohol use, n (%)	Hazardous drinking, n (%)	Harmful drinking, n (%)	Possible alcohol dependence, n (%)
Overall		167 (66.8)	69 (27.6)	9 (3.6)	5 (2.0)
Sex	Female	23 (65.7)	7 (20.0)	3 (8.6)	2 (5.7)
	Male	144 (67.0)	62 (28.8)	6 (2.8)	3 (1.4)
Age (years)	18–20	21 (65.6)	8 (25.0)	2 (6.3)	1 (3.1)
	21–25	55 (68.8)	24 (30.0)	–	1 (1.3)
	26–34	47 (68.1)	16 (23.2)	5 (7.2)	1 (1.4)
	35–45	35 (14.0)	16 (6.4)	1 (0.4)	2 (0.8)
	46 and above	9 (60.0)	5 (33.3)	1 (6.7)	–
Marital status	Single	93 (66.9)	37 (26.6)	6 (4.3)	3 (2.2)
	Married	74 (67.9)	31 (28.4)	2 (1.8)	2 (1.8)
	Divorced	–	1 (50.0)	1 (50.0)	–
Force branch	Air Force	11 (68.8)	5 (31.2)	–	–
	Ground Force	156 (66.8)	69 (27.6)	9 (3.6)	5 (2.0)
Military rank	NCO and lower ranks	141 (66.5)	57 (26.9)	9 (4.2)	5 (2.4)
	Commissioned officer	26 (68.4)	12 (31.6)	–	–

AUDIT, Alcohol Use Disorder Identification Test; NCO, non-commissioned officer.

### Multivariable analysis

In the final regression model, no statistically significant differences emerged from education. Marital status, military rank and depression levels by drinking in the cross-tabulation analysis were dropped from the multivariable regression analysis due to their p values of ≥0.25. Drinking alcohol was found to be significantly associated with sex, age, force branch, smoking status and risk-taking propensity. Those under 35 years of age had around 50% protection against drinking alcohol compared with those 35 years or older. Men had 1.7 times higher odds of drinking alcohol compared with women. Ground Force respondents had around fourfold odds of drinking alcohol compared with Air Force personnel. Smokers had more than sixfold odds of drinking compared with non-smokers. In addition, those categorised under the high risk-taking propensity category had twofold odds of drinking alcohol compared with those categorised under the low risk-taking propensity category (Table 4).

**Table 4:** Binary logistic regression of sociodemographic characteristics and other factors by current drinking among military personnel (n=220)

Variable	Category	Current drinking		COR* (95% CI)	AOR* (95% CI)
		No, n (%)	Yes, n (%)		
Sex	Female	54 (60.7)	35 (39.3)	1	1
	Male	198 (47.9)	215 (52.1)	0.597 (0.374 to 0.952)	1.710 (1.011 to 2.893)*
Age (years)	< 35	207 (52.9)	184 (47.1)	1.650 (1.076 to 2.531)	0.522 (0.316 to 0.861)*
	≥ 35	45 (40.5)	66 (59.5)	1	1
Marital status	Single	149 (51.9)	138 (48.1)	1.177 (0.824 to 1.681)	
	Married	100 (47.8)	109 (52.2)	1	
Education	High school or less	185 (46.6)	212 (53.4)	0.495 (0.317 to 0.772)	1.158 (0.655 to 2.049)
	Some college and above	67 (63.8)	38 (36.2)	1	1
Force branch	Air Force	62 (79.5)	16 (20.5)	1	1
	Ground Force	190 (44.8)	234 (55.2)	4.772 (2.667 to 8.540)	3.877 (1.861 to 8.078)**
Military rank	NCO* and lower ranks	208 (49.5)	212 (50.5)	1.180 (0.734 to 1.896)	
	Commissioned officer	44 (53.7)	38 (46.3)	1	
Smoking	No	246 (53.1)	217 (46.9)	1	1
	Yes	6 (15.4)	33 (84.6)	6.235 (2.584 to 15.165)	6.254 (2.499 to 15.651)**
Depression level	Minimal	68 (43.9)	87 (56.1)	1	
	Mild and moderate	26 (38.8)	41 (61.2)	1.233 (0.687 to 2.212)	
Risk-taking propensity	Low	141 (64.7)	77 (35.3)	1	1
	High	108 (38.6)	172 (61.4)	2.916 (2.019 to 4.211)	2.347 (1.568 to 3.514)**

*P<0.05, **P<0.01.

AOR, adjusted OR; COR, crude OR; NCO, non-commissioned officer.

## Discussion

The principal findings of this study were the alcohol use levels of current drinkers, hazardous and harmful alcohol use, and its associated factors in Ethiopian military personnel. Half of participants reported current alcohol use, of which 63.1% were infrequent users, 26.7% were moderate drinkers and 10.2% were heavy drinkers. Another important finding was that 33% of men and 25% of women met criteria indicative of hazardous and harmful alcohol use and possible alcohol dependence. The current study documented that alcohol drinking was found to be statistically significantly associated with sex, age, force branch, smoking and risk-taking. Our discussions of these findings are presented further.

The finding of current alcohol use in this study was compared with those of sub-Saharan African countries’ studies. The finding of this study was consistent with that of a Nigerian Army study (53.9%), despite the fact that only military officers were included,^12^ but lower compared with finding from the Angolan Armed Forces (60%),^11^ and the Congo Armed Forces (77.2%) studies.^10^ These differences may be attributable to study design: the Angolan Armed Forces study was longitudinal, and the Congo Armed Forces study was a secondary analysis of data that included only participants who reported ever having sex. Women were excluded from both studies. Moreover, the finding of this study was lower compared with findings from the Sri Lanka military study (71.2%).^9^ A possible explanation for this might be that the Sri Lanka study was focused exclusively on the Navy. Studies indicate higher alcohol consumption among naval personnel^23 24^; the Navy Forces could have a higher physical and social availability of alcohol.^25^ Women were excluded from the Sri Lanka study, and women in the military drank less than their men counterparts,^26^ perhaps due to more combat duty for men.^27 28^ The current study level of infrequent drinkers was higher compared with Sri Lankan military forces (54.81%).^9^ It seems possible that this difference might be due to drinking culture.

The current study finding that men participants met criteria indicative of hazardous and harmful alcohol use and possible alcohol dependence was higher compared with the findings of the Sri Lanka military forces study (16.69%)^9^ and the Armed Forces of the Republic of the Congo study (15.8%).^10^ This discrepancy might be due to some restrictions on alcohol availability in the military camps of the Sri Lanka Army, and in the Congo Armed Forces study, study participants were married. The current study finding was consistent with the Angolan Armed Forces study (35%).^11^ However, this study finding was lower compared with the Botswana Defence Force study (58.9%).^17^ A possible explanation for this difference could be that the Botswana Defence Force study focused on personnel aged 18–30 years. In accordance with the present study of women, the finding was higher compared with that of the Malawi Defence Force (3.8%).^16^ This discrepancy might be due to the invitation extended to all women service members to participate in the Malawi study.

In the present study, factors associated with alcohol use were sex, age, force branch, smoking and risk-taking. The findings of this study were in line with a US military study^29^ in the case of younger age and male sex as associated factors. However, the age findings contradict the Angolan soldiers’ study that found being older as a risk factor.^11^

A key strength of the present study was the active participation of the study participants. The other strength was that the alcohol use assessment was done using a well-validated, widely used measure.

This study also had limitations. Although respondents were assured of confidentiality, reliance on self-reporting may have resulted in an underestimation of alcohol consumption as a result of social desirability bias and should be interpreted with caution. Participants may be prone to recall errors when reporting alcohol use in the past week and during the past 12 months. This study looks only at alcohol consumption at one time point because of its cross-sectional design, prohibiting us from evaluating the impact of hazardous alcohol consumption on health. The current study did not consider the lifetime prevalence of alcohol use.

## Conclusions

The results of this study indicate that for Ethiopian military personnel, the level of alcohol use, hazardous alcohol consumption and the association between smoking, high risk-taking and alcohol may need further attention. These findings highlight the need for additional research into the factors that contribute to problematic alcohol use, as well as the possibility of further studies and prevention programmes targeting the alcohol spectrum, which ranges from abstinence and low-risk use to more severe alcoholism and alcohol dependence. Findings from this study could help improve policy and provide insight into developing effective alcohol intervention programmes for military personnel not only in Ethiopia but also in other resource-limited countries.

## Data Availability

Data are available upon reasonable request.
